# Rapid early progression (REP) of glioblastoma is an independent negative prognostic factor: Results from a systematic review and meta-analysis

**DOI:** 10.1093/noajnl/vdac075

**Published:** 2022-06-04

**Authors:** Mueez Waqar, Federico Roncaroli, Eric J Lehrer, Joshua D Palmer, Javier Villanueva-Meyer, Steve Braunstein, Emma Hall, Marianne Aznar, Philip C De Witt Hamer, Pietro I D’Urso, Daniel Trifiletti, Alfredo Quiñones-Hinojosa, Pieter Wesseling, Gerben R Borst

**Affiliations:** Department of Neurosurgery, Geoffrey Jefferson Brain Research Centre, Salford Royal NHS Foundation Trust, Manchester, UK; Division of Cancer Sciences, Faculty of Biology, Medicines and Health, The University of Manchester, Manchester, UK; Neuropathology unit, Geoffrey Jefferson Brain Research Centre, Salford Royal NHS Foundation Trust, Manchester, UK; Division of Neuroscience and Experimental Psychology, Faculty of Biology, Medicines and Health, The University of Manchester, Manchester, UK; Department of Radiation Oncology, Icahn School of Medicine at Mount Sinai, New York, USA; Division of Neuroscience and Experimental Psychology, Faculty of Biology, Medicines and Health, The University of Manchester, Manchester, UK; Department of Radiation Oncology, Icahn School of Medicine at Mount Sinai, New York, USA; Department of Radiation Oncology, The James Cancer Hospital, Ohio, USA; Department of Neuroradiology, University of California San Francisco, San Francisco, USA; Department of Radiation Oncology, University of California San Francisco, San Francisco, USA; Division of Cancer Sciences, Faculty of Biology, Medicines and Health, The University of Manchester, Manchester, UK; Division of Cancer Sciences, Faculty of Biology, Medicines and Health, The University of Manchester, Manchester, UK; Department of Neurosurgery, Amsterdam University Medical Centers/VUmc, Amsterdam, The Netherlands; Department of Neurosurgery, Geoffrey Jefferson Brain Research Centre, Salford Royal NHS Foundation Trust, Manchester, UK; Department of Radiation Oncology, Mayo Clinic, Jacksonville, Florida, USA; Department of Neurosurgery, Mayo Clinic, Jacksonville, Florida, USA; Department of Pathology, Amsterdam University Medical Centers/VUmc, Amsterdam, The Netherlands; Laboratory for Childhood Cancer Pathology, Princess Máxima Center for Pediatric Oncology, Utrecht, The Netherlands; Division of Cancer Sciences, Faculty of Biology, Medicines and Health, The University of Manchester, Manchester, UK; Department of Radiation Oncology, The Christie NHS Foundation Trust, Manchester, UK; Department of Radiotherapy Related Research, The Christie NHS Foundation Trust, The Christie National Health Trust, Manchester, UK

**Keywords:** extent of resection, glioblastoma, IDH, MGMT, prognosis, progression, recurrence, REP, survival

## Abstract

**Background:**

In patients with newly diagnosed glioblastoma, rapid early progression (REP) refers to tumor regrowth between surgery and postoperative chemoradiotherapy. This systematic review and meta-analysis appraised previously published data on REP to better characterize and understand it.

**Methods:**

Systematic searches of MEDLINE, EMBASE and the Cochrane database from inception to October 21, 2021. Studies describing the incidence of REP—tumor growth between the postoperative MRI scan and pre-radiotherapy MRI scan in newly diagnosed glioblastoma were included. The primary outcome was REP incidence.

**Results:**

From 1590 search results, 9 studies were included with 716 patients. The median age was 56.9 years (IQR 54.0–58.8 y). There was a male predominance with a median male-to-female ratio of 1.4 (IQR 1.1–1.5). The median number of days between MRI scans was 34 days (IQR 18–45 days). The mean incidence rate of REP was 45.9% (range 19.3%–72.0%) and significantly lower in studies employing functional imaging to define REP (*P* < .001). REP/non-REP groups were comparable with respect to age (*P* = .99), gender (*P* = .33) and time between scans (*P* = .81). REP was associated with shortened overall survival (HR 1.78, 95% CI 1.30–2.43, *P* < .001), shortened progression-free survival (HR 1.78, 95% CI 1.30–2.43, *P* < .001), subtotal resection (OR 6.96, 95% CI 4.51–10.73, *P* < .001) and IDH wild-type versus mutant tumors (OR 0.20, 95% CI 0.02–0.38, *P* = .03). *MGMT* promoter methylation was not associated with REP (OR 1.29, 95% CI 0.72–2.28, *P* = .39).

**Conclusions:**

REP occurs in almost half of patients with newly diagnosed glioblastoma and has a strongly negative prognostic effect. Future studies should investigate its biology and effective treatment strategies.

**Key Points**

- Rapid early progression (REP) occurs in almost half of all newly diagnosed glioblastoma patients.
- REP is a strongly negative prognostic factor in newly diagnosed glioblastoma.

**Importance of the Study**

This meta-analysis demonstrates that almost half of all patients with newly diagnosed glioblastoma experience tumor regrowth referred to as rapid early progression (REP) in the time interval between surgery and postoperative chemoradiotherapy. REP is a strongly negative prognostic factor and associated with decreased overall and progression-free survival. However, it remains poorly understood and future studies are needed to better understand its biological basis to develop effective treatment strategies.

Glioblastoma is the most common primary intrinsic brain tumor in adults and it is associated with a dismal prognosis. Despite the best treatment modalities comprising maximal safe resection and postoperative chemoradiotherapy, the median survival remains between 15 and 21 months and <5% of patients survive more than 5 years.^1–3^ The 5-year survival has not changed significantly for decades.^4^

For newly diagnosed glioblastoma, it is often quoted that the median time to recurrence after postoperative adjuvant therapy is 7 months.^1^ However, the first evidence of tumor progression may occur prior to commencement of postoperative chemoradiotherapy. This first and initial tumor progression in the time interval between surgery and postoperative adjuvant therapy is often referred to as Rapid Early Progression (REP; Figure 1).

**Figure 1.**
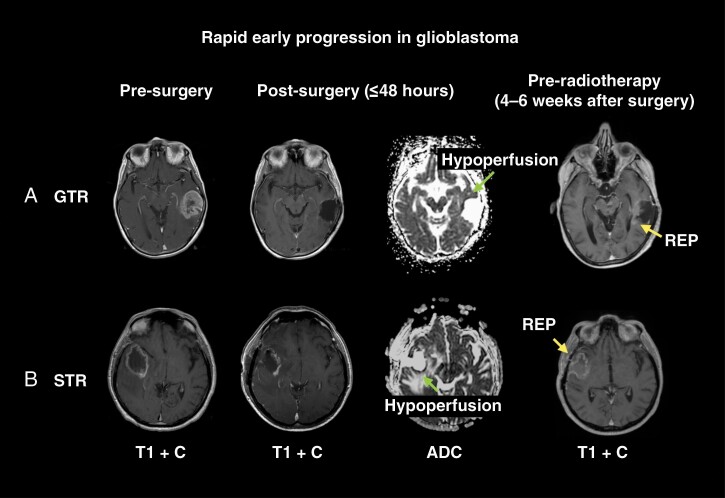
Example of rapid early progression (REP) in newly diagnosed glioblastoma. This figure shows serial MRI scans during various treatment time points of 2 patients with newly diagnosed glioblastoma to highlight the occurrence of REP after both gross-total resection (GTR) and subtotal resection (STR). (A) A 76-year-old female who was normally fit and well presented with dysphasia and was found to have a large left temporal tumor. She underwent a craniotomy and GTR of the tumor. Histology was consistent with an IDH wild-type glioblastoma. The pre-radiotherapy MRI scan demonstrated a modest volume of REP (white arrow) that was separate to the areas of restricted diffusion indicative of surgical cavity hypoperfusion (black arrow on the apparent diffusion coefficient [ADC] scan) on the post-operative MRI scan. (B) A 47-year-old female presented with 3 weeks of progressively worsening headaches and an MRI scan revealed a right posterior temporal tumor. She underwent a craniotomy and subtotal resection of the tumor. Histology was consistent with an IDH-wild-type glioblastoma. The pre-radiotherapy MRI scan demonstrated a large volume of REP (white arrow) that was distinct to the areas of restricted diffusion indicative of surgical cavity hypoperfusion (black arrow on ADC scan) on the post-operative MRI scan. Abbreviations: T1 + C = T1 with contrast; ADC = apparent diffusion coefficient. Pre-op = Preoperative; Post-op = Postoperative.

Pirzkall et al. were the first to describe the incidence of REP. In a series of 32 patients with newly diagnosed glioblastoma, 17 (53%) had new areas of enhancement suspicious for tumor growth on the pre-radiotherapy MRI scan compared with the immediate postoperative MRI scan, separated by an average of 27 days. REP was found to be associated with decreased overall survival.^5^ Since this study, several other reports have described the incidence of REP, and it appears to be common even after gross-total resection (GTR).^6,7^

REP may have future implications for glioblastoma treatment and could indicate a subset of patients with intrinsically more aggressive tumors that may benefit from more intensified/targeted upfront therapy. To date, REP has not been subject to extensive investigation. The aim of this systematic review and meta-analysis was to pool available published data on REP in glioblastoma to better characterize and understand it.

## Methods

### Registration

The study protocol was registered on the international prospective register of systematic reviews (PROSPERO) under the ID number: CRD42022301242. The review was undertaken, and the manuscript prepared according to PRISMA (Preferred Reporting Items for Systematic Reviews and Meta-Analysis; see Supplementary Material) and MOOSE guidelines (see Supplementary Material).

## Literature Search

The literature search strategy is outlined in Supplementary Table 1. All searches were conducted by two independent authors (MW and EH). MEDLINE, EMBASE, and the Cochrane Database of Systematic Reviews were queried from inception to October 21, 2021 using the NICE Healthcare Databases Advanced Search (HDAS) service. References of included studies were examined to extract potential further papers that may have been missed during the initial systematic search. Two independent authors (MW and EH) screened titles and abstracts independently and blindly to identify articles meeting the inclusion criteria. Discrepancies were resolved through discussion and review by a third author (GB). Studies were carefully screened and all duplicates were removed.

## Article Inclusion

Articles comparing the first postoperative MRI scan and pre-radiotherapy MRI scan in glioblastoma patients were included. This comparison involved structural and/or functional imaging (see Outcomes). A PICOS table is provided in Supplementary Table 2.

## Data Extraction

Data relating to patient demographics, number of days between the two MRI scans, extent of resection, MRI scan type, *MGMT* promoter methylation, IDH mutation status, and REP were extracted into a Microsoft Excel spreadsheet. Hazard ratios relating to overall survival (OS) and progression-free survival (PFS) were also collected together with confidence intervals.

## Outcomes and Definitions

1. Primary: incidence rate of REP—defined as interval tumor growth between the postoperative and pre-radiotherapy MRI scans. In general, tumor growth referred to an increase in enhancing tumor between these time points.
2. Secondary:
3. Impact of demographic factors and time between MRI scans on REP.
4. Impact of type of MRI scan sequences used to assess REP: the rate of REP was compared between studies utilizing structural MRI sequences alone (eg T1 with contrast; denoted as the structural group) versus those using structural plus one or more functional sequences (e.g. diffusion and/or perfusion-weighted MRI—DWI/PWI; denoted as the functional group).
5. Impact of REP on overall survival (OS).
6. Impact of REP on the location of future disease progression and progression-free survival (PFS). PFS was defined in standard terms as the time interval between surgery and first evidence of disease progression after postoperative chemoradiotherapy (ie not including REP).
7. Impact of extent of resection on REP: the rate of REP was compared between gross total-resection (GTR) versus subtotal resection (STR)/biopsy. The latter two groups were combined given the sample size of the biopsy group. The study-specific definition of GTR was used.
8. Impact of MGMT promoter methylation and IDH mutation status on REP.

## Risk of Bias

As all included studies were diagnostic and non-randomized, the risk of bias was assessed using the QUADAS-2 tool.^8^ This is designed for diagnostic studies and recommended by the Cochrane group.^9^

## Statistical Analysis

Statistical analysis was performed in R version 4.0.5 (R Foundation for Statistical Computing; Vienna, Austria). Baseline factors were compared between REP/non-REP groups using nonparametric weighted Mann–Whitney U tests by sample size, given the paucity of reported variance, and Fisher’s exact tests. Demographic factors were described with weighted medians and interquartile ranges (IQR) or ranges, with an indicator of the number of studies used. All meta-analyses were performed using the Meta package in R using the Cochrane Revman template. Dichotomous outcome meta-analyses were performed using a Mantel–Haenszel method and fixed- or random-effects models, using odds ratios or risk difference if the event rate was zero in one group. Generic inverse variance meta-analyses were used for survival analyses with hazard ratios. Fixed-effects models were used when interstudy heterogeneity was judged as low, otherwise random-effects models were used. Sensitivity analyses were performed using a subset of studies assessing REP using functional imaging.

## Results

From 1590 search results, 836 unique records were found and 9 studies were included in quantitative meta-analysis (Supplementary Figure 1).^5–7,10–15^ Study characteristics are presented in Table 1, including 7 retrospective and 2 prospective studies. Patient selection accounted for the highest source of bias (56%) (Supplementary Table 3 and Supplementary Figure 2).

**Table 1.** Studies Investigating Rep in Glioblastoma. 9 Studies were Included in Quantitative Meta-Analysis Across 7 Countries. Some Studies Used Only Structural Imaging to Define Rep Whereas Others Additionally Used One or More Functional Imaging Sequences

Study and institute	Design	MRI sequences used to define REP	REP definition	Total N
**Lakomy 2020** Brno, Czech Republic	Retrospective case series	T1 + C	≥25% increase in enhancing residuum OR new enhancing lesion OR unambiguous progression	90
**Palmer 2019** Ohio, USA	Retrospective case series	T1 + C	Increase in nodular enhancement OR new satellite lesion OR ≥25% increase in residuum	87
**De Barros 2019** Toulouse, France	Retrospective case series	T1 + C, DWI	New enhancement without restricted diffusion around surgical cavity	75
**Merkel 2017** Erlangen, Germany	Retrospective case series	T1 + C	Increase in nodular enhancement at border of cavity OR new satellite lesion OR increase in residuum	61
**Wee 2017** Seoul, Korea	Retrospective case series	T1 + C, DWI, PWI	Increase in ≥25% of enhancing residual OR new enhancing lesion with increased blood flow + diffusion restriction (separate from surgical cavity)	166
**Villanueva‐Meyer 2017** San Francisco, USA	Retrospective case series	T1 + C, DWI	New enhancement without restricted diffusion	140
**Majos 2016** Valles, Spain	Prospective case series	T1 + C	New enhancement*	28
**Farace 2013** Verona, Italy	Retrospective case series	T1 + C, DWI, PWI	New enhancement, without restricted diffusion OR with high perfusion	37
**Pirzkall 2008** San Francisco, USA	Prospective case series	T1 + C, DWI	New enhancement not entirely related to area of restricted diffusion	32

*This study’s definition of REP included only those cases that demonstrated progression on subsequent follow-up imaging, but all cases with change on the interval MRI were included for comparative purposes. Abbreviations: T1 + C = T1 with contrast MRI; DWI = diffusion-weighted MRI; PWI = perfusion-weighted MRI.

In total, 716 patients with newly diagnosed glioblastoma were included across all studies from 7 different countries. The median age was 56.9 years (IQR 54.0–58.8 years; 6 studies). There was a male excess with a median male-to-female ratio of 1.4 (IQR 1.1–1.5; 5 studies). The median interval between the postoperative MRI scan and pre-radiotherapy MRI scan was 34 days (IQR 18–45 days; 9 studies). The indication for the pre-radiotherapy MRI scan was not detailed in included studies. A total of 485 patients across 6 studies had data pertaining to the extent of resection, although this was only explicitly defined in 4 studies. In all 4 studies, GTR was defined as the absence of postoperative residual enhancing disease. The breakdown of extent of resection was as follows: GTR 184/485 (37.9%), STR 272/485 (56.1%), and biopsy 29/485 (6.0%). No patient was reported to undergo reoperation for STR.

## Primary Outcome

**Figure 2.**
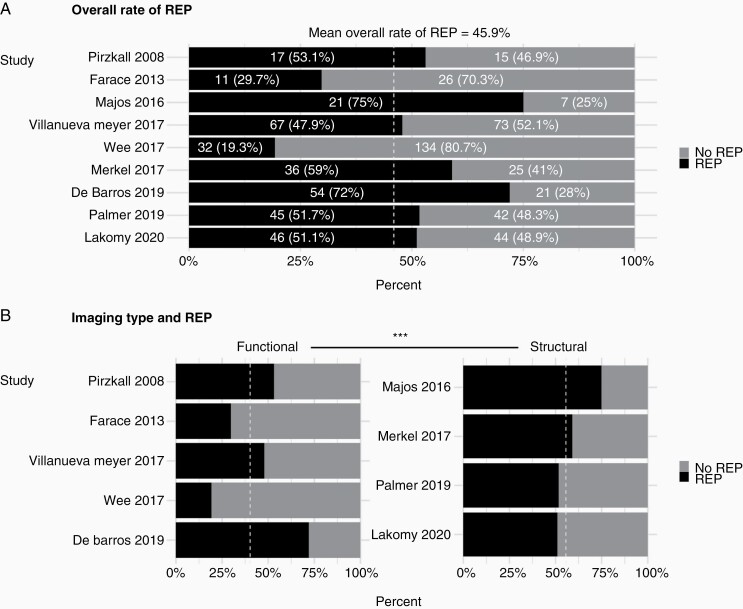
Incidence rate of REP in glioblastoma. (A) This figure shows proportions and percentages of patients with/without REP across studies to demonstrate the overall mean rate of REP (45.9%). (B) This figure divides studies into 2 groups based on the use of functional MRI to define REP. Studies employing functional imaging reported a significantly lower mean incidence rate of REP (55.6% vs. 40.2%, Fisher’s exact, *P* < .001).

The overall mean incidence rate of REP was 45.9% (reported range 19.3%–72.0%; Figure 2A). Data relating to the location of REP were available in 103 patients across 3 studies. The vast majority (85/103, 82.5%) of REP lesions were within or adjacent to the surgical cavity and only a minority was described as de novo and distant from the surgical cavity (18/103, 17.5%).

## Secondary Outcomes

A comparison between patients with and without REP revealed that there were no significant differences in mean age (57 vs. 57 years, Mann-Whitney, *P* = .62), gender ratio (%males 57.6% vs. 62.9%, Fisher’s Exact, p*P* = .33) or mean time between MRI scans (30.5 vs. 29.6 days, Mann-Whitney, *P* = .27) between these groups.

Studies using functional imaging sequences to define REP (Table 1) did not consider all enhancing signal as indicative of tumor regrowth. Therefore, the overall mean incidence rate of REP was significantly higher in studies employing structural imaging alone to define REP versus those using structural and one or more functional imaging sequences (55.6% vs. 40.2%, Fisher’s Exact, *P* < 0.001; Figure 2B).

**Figure 3.**
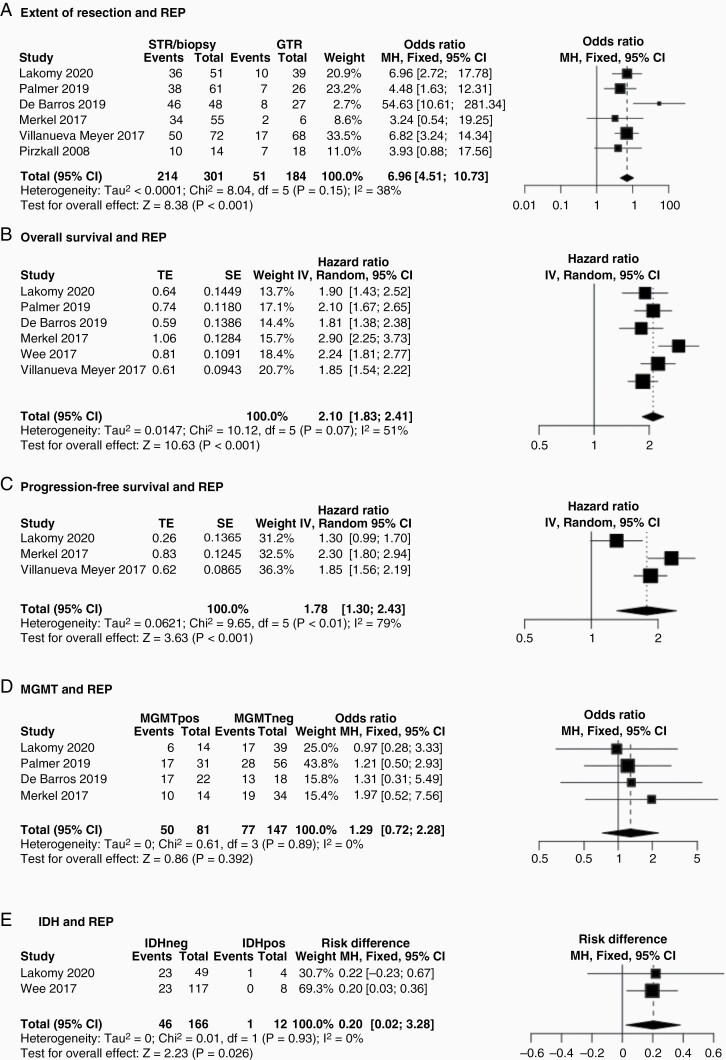
Associations of REP. (A) Comparison of patients with gross total versus subtotal resection/biopsy (combined as the biopsy group was relatively small). Subtotal resection/biopsy were predictive of REP using a fixed-effects model (OR 6.96, 95% CI 4-51-10.73, *P* < .001). (B) Patients with REP had a higher hazard ratio of death (HR 2.10, 95% CI 1.83–2.41, *P* < .001) using a random-effects model. (C) Patients with REP had a higher hazard ratio of disease progression (HR 1.78, 95% CI 1.30–2.43, *P* < .001) using a random-effects model. (D) *MGMT* promoter methylation (denoted as “MGMTpos” versus “MGMTneg” to denote its absence) was not associated with REP incidence (OR 1.29, 95% CI 0.72–2.28, *P* = .39) using a fixed-effects model. (E) Only 2 studies presented data on the interrelations between REP and IDH. IDH wild-type tumors (IDHneg) had a significantly higher incidence of REP versus IDH mutant (IDHpos) tumors (OR 0.20, 95% CI 0.02–0.38, *P* = .03) using a fixed-effects model.

**Figure 4.**
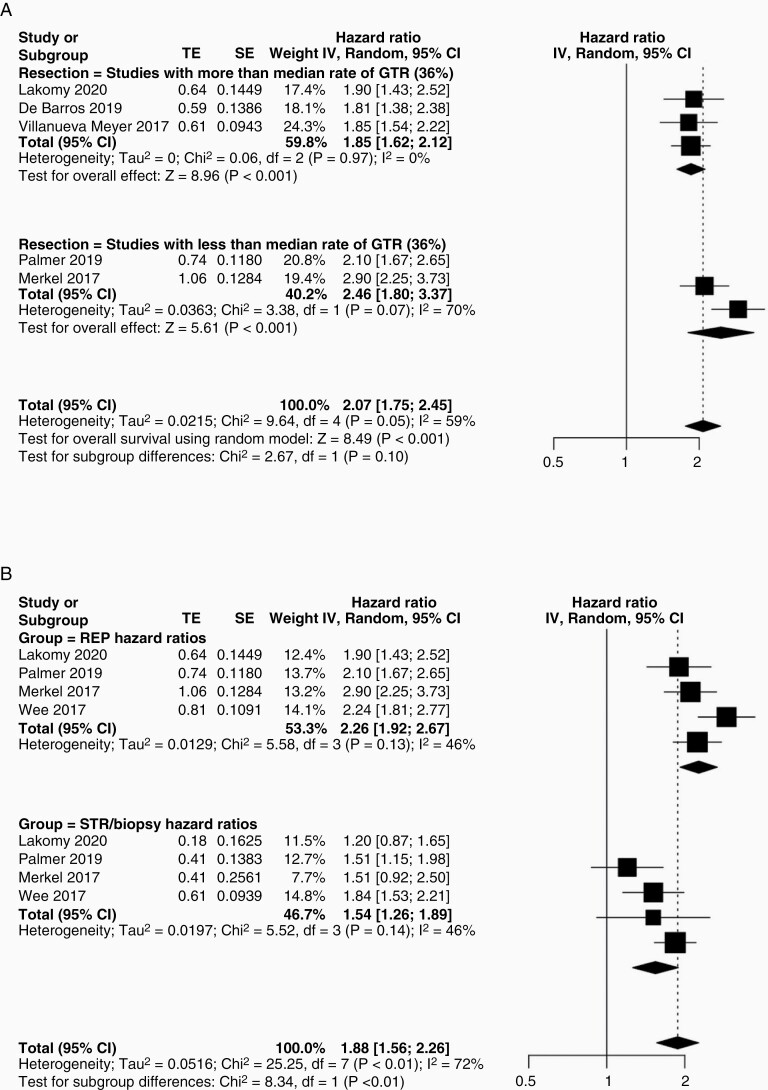
Subtotal resection and REP—subgroup analyses. (A) Studies were stratified into 2 groups based on the median rate of GTR and the group with a lower rate of GTR had a tendency toward a higher incidence rate of REP, although differences were not statistically significant (OR 2.07, 95% CI 1.75–2.45, χ ^2^ = 2.67, *P* = .10). (B) To determine which factor may have a bigger effect on prognosis, we pooled and compared hazard ratios for STR/biopsy versus REP in 4 studies that presented this comparative data. REP was associated with a significantly higher overall hazard ratio of death (χ ^2^ = 8.34, *P* < .01).

Meta-analysis results are presented in Figures 3–4. Subtotal resection/biopsy were predictive of REP (OR 6.96, 95% CI 4.51–10.73, *P* < .001; Figure 3A). REP was an independent negative prognostic factor and associated with OS due to an increased hazard ratio of death (HR 2.10, 95% CI 1.83–2.41, *P* < .001; Figure 3B). REP was also associated with PFS due to an increased hazard ratio of post-radiotherapy disease progression (See Methods for REP/PFS definitions; HR 1.78, 95% CI 1.30–2.43, *P* < .001; Figure 3C). *MGMT* promoter methylation was not associated with REP incidence (50/81 vs. 77/147; OR 1.29, 95% CI 0.72–2.28, *P* = .39; Figure 3D). IDH wildtype tumors had a significantly higher incidence of REP versus IDH mutant tumors (46/166 vs. 1/12; OR 0.20, 95% CI 0.02–0.38, *P* = .03; Figure 3E), even though this was only assessed in two studies.

One study robustly evaluated the correlation between the location of REP and future disease progression in 42 patients. In the vast majority of these patients (39/42, 92.9%), further disease progression occurred at sites of REP.^7^

Given the association of REP with both extent of resection and OS, we further analyzed the interrelations between these variables (Figure 4). Five studies provided sufficient data to allow binary study subgrouping based on the median rate of GTR (36.0%; Figure 4A). Studies with less than the median rate of GTR had a higher incidence rate of REP although differences did not reach statistical significance (OR 2.07, 95% CI 1.75–2.45, Chi-squared = 2.67, *P* = .10; Figure 4A). We then compared the effect size of STR/biopsy versus REP on overall survival by comparing pooled hazard ratios in 4 studies providing data for these variables. REP was associated with a significantly higher overall hazard ratio of death (Chi-squared = 8.34, *P* < .01; Figure 4B).

## Publication Bias

Funnel plots were derived to evaluate for publication bias (Supplementary Figure 3). A visual inspection of funnel symmetry was undertaken due to the paucity of studies to allow an adequately powered regression analysis. Publication bias was evident for extent of resection (Supplementary Figure 3A), but not for other outcomes.

## Sensitivity Analysis

Sensitivity analyses were possible for extent of resection and OS (Supplementary Figure 4). Both of these associations were present as noted above in the subset of studies using functional imaging to define REP.

## Discussion

In this systematic review and meta-analysis, we demonstrated that REP occurs in almost half of all newly diagnosed glioblastoma patients, usually within or adjacent to the surgical cavity. Functional imaging more accurately defines REP than structural imaging. REP was significantly more likely after STR and in IDH wild-type versus IDH mutant glioblastomas, but was not influenced by *MGMT* status. REP was an independent negative prognostic factor, associated with decreased OS and earlier disease progression, most often representing the site of future progression. The impact of REP on survival was significantly greater than STR.

A considerable proportion of glioblastoma patients experienced REP, although there was large variation in reported incidence rates. Although imaging modalities can account for some of this variation, it is not the only explanation as the reported rate of REP in studies employing one or more functional imaging sequences to define REP ranged from 19.3% to 72.0%.^11,12^ The former incidence was reported by Wee et al. who applied the most stringent definition of REP requiring two functional imaging sequences (DWI and PWI); the latter incidence was reported by De Baros et al. who only utilized DWI.^11,12^ Surgery is known to induce hypoperfusion of tissue adjacent to the surgical cavity that initially results in restricted diffusion and subsequently enhancement with contrast during short interval follow-up.^16^ It is for this reason that postoperative imaging within 48 hours is recommended to assess residual disease.^17^ Surgical cavity hypoperfusion is distinct from REP, although its increased incidence in patients with STR may suggest that it is in part driven by the biological effects of postoperative hypoxia on greater tumor residuum. These can include pro-tumorigenic induction of tumor cells to a more stem cell-like phenotype and increased discordant angiogenesis.^18^ The subsequent leaky vessels in more proliferative tumor beds could explain the interval increase in contrast uptake.

The high incidence of REP supports a routine multi-modal pre-radiotherapy MRI scan in glioblastoma patients, which includes at least one functional imaging sequence to mitigate for the effects of surgery. This was not the practice at any of the studies included in this review and contrary to current guidance.^17^ Given the correlation between REP sites and future disease progression, omitting this scan may grossly underestimate the required irradiation volume. The use of functional imaging sequences as part of this scan is justified as above, but notably, functional imaging sequences have limited voxel resolutions that may not detect very small tumor residuum.^19^

Our analysis demonstrated that REP is clinically relevant and not just related to remnant enhancing tumor, as patients with GTR can also develop REP. These patients harbor a variable volume of non-enhancing disease diffusely infiltrating the brain that may play a role in REP.^20^ The relationship between a greater enhancing tumor residuum and increased incidence of REP supports the standard of maximal safe resection in glioblastoma patients. Indeed, Wee et al. reported that every 1 cm^3^ increase in residual enhancing disease increased the risk of REP by 3.9%.^12^ However, preclinical studies have demonstrated that mechanical cell injury as induced by surgery can increase tumor cell migration, proliferation, and infiltration.^21,22^ These effects can be considerable and contribute, for example, to dynamic growth of the biopsied component of a multi-focal glioblastoma versus the non-biopsied component.^21^ These observations require further validation in relation to the beneficial effects of surgery. In addition, the biological basis for REP remains unclear and has not been investigated at the histological or molecular level due to the rarity of reoperation for glioblastoma.^23^

The frequency of REP may actually be under-represented here given that not all patients make it to postoperative adjuvant therapy and represents an important challenge to the treatment strategy for newly diagnosed glioblastoma.^24^ At present, this comprises surgery followed by chemoradiotherapy after a time period of 4–6 weeks. In this early time period (ie pre-, intra-, or early-postoperative period), intensified upfront therapy could counteract factors contributing to REP as simply commencing postoperative adjuvant therapy earlier does not improve outcome.^25–27^ We recently reviewed these early treatment strategies, and found that neoadjuvant immunotherapy and intraoperative radiotherapy may represent the most promising options.^28–30^ Early intensified therapy has demonstrated benefit in several other cancer types. For example, neoadjuvant chemotherapy/radiotherapy can downstage locally advanced breast cancer, sarcoma, and several gastrointestinal cancers, improving the likelihood of organ preserving gross total resection.^31–34^ Our results highlight the importance of further investigation of early interventions for newly diagnosed glioblastoma.

Although our review evaluated the association between REP and several factors, existing data are not exhaustive and REP remains relatively understudied. No study has related the preoperative growth rate of glioblastoma to REP, which would otherwise control for the major confounder of the tumor’s intrinsic aggressiveness. Another important question that requires further investigation is which postoperative residual glioblastoma niches contribute to REP. Macroscopically, factors such as necrosis, vascularity, and prognostically unfavorable tumor locations (eg adjacent to the subventricular zone, SVZ) could be related and should be studied in future. SVZ adjacency (<5 mm) was evaluated in a single study of 75 glioblastoma patients in which it was not associated with REP in multivariate analysis.^11^

Limitations of this meta-analysis include the retrospective nature of many studies and the different definitions of REP. There was a high risk of bias relating to patient selection and incomplete imaging at the time points of interest. However, the overall sample size was relatively large and results were consistent/comparable between studies. Data relating to the indication for the pre-radiotherapy MRI scan were not available, although this is often routinely performed in centers such as our own. Not all studies presented data pertaining to secondary outcomes, so this analysis was subject to publication bias. Not enough studies presented data on patient treatment, including the use of concurrent medication such as corticosteroids, which could have limited our analysis. Studies also did not describe the clinical impact of REP on patient management. Lastly, there was also a uniform lack of investigation of the biological basis of REP.

## Conclusion

This systematic review and meta-analysis demonstrates that almost half of all patients with newly diagnosed glioblastoma experience tumor recurrence referred to as REP in the time interval between surgery and postoperative chemoradiotherapy. REP has a strongly negative prognostic effect on both OS and PFS, and is more common in patients with a STR. Its effect on prognosis appears to be even worse than STR. The biological basis of REP remains unclear and should be subject to future investigation through development of REP prediction models as well as prospective validation in patients. The high incidence of REP should also encourage efforts to better understand the role of early intensified therapy for glioblastoma.

## Supplementary Material

vdac075_suppl_Supplementary_Figures

vdac075_suppl_Supplementary_Tables
