# Erratum to: Rapid early progression (REP) of glioblastoma is an independent negative prognostic factor: Results from a systematic review and meta-analysis

**DOI:** 10.1093/noajnl/vdac125

**Published:** 2022-08-09

This is an erratum to: Mueez Waqar, Federico Roncaroli, Eric J Lehrer, Joshua D Palmer, Javier Villanueva-Meyer, Steve Braunstein, Emma Hall, Marianne Aznar, Philip C De Witt Hamer, Pietro I D’Urso, Daniel Trifiletti, Alfredo Quiñones-Hinojosa, Pieter Wesseling, Gerben R Borst, Rapid early progression (REP) of glioblastoma is an independent negative prognostic factor: Results from a systematic review and meta-analysis, Neuro-Oncology Advances, Volume 4, Issue 1, January-December 2022, vdac075, https://doi.org/10.1093/noajnl/vdac075

In the originally published version of this manuscript, the surname of author Gerben R Borst was inadvertently misspelled as Gerben R Bors.

This error has been corrected.

