# Supplementary material for: Rapid early progression (REP) of glioblastoma is an independent negative prognostic factor: Results from a systematic review and meta-analysis
Source: Neurooncol Adv. 2022 Jun 4;4(1):vdac075. doi: 10.1093/noajnl/vdac075 (PMC9234755; doi:10.1093/noajnl/vdac075)

**Supplementary Figures**

**Supplementary Figure 1. PRISMA diagram indicating literature screening and study inclusion**.


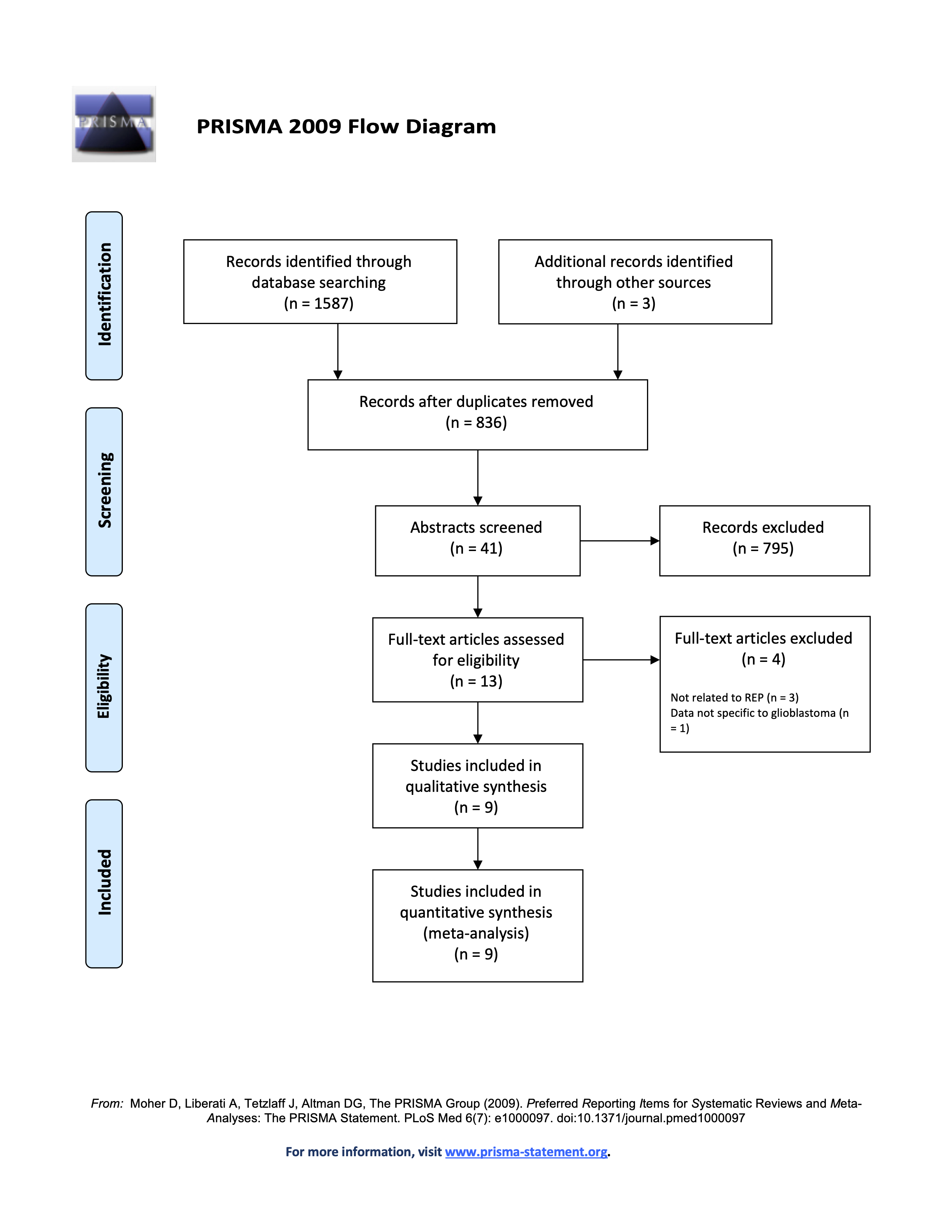


**Supplementary Figure 2. Risk of bias assessment using the QUADAS-2 tool**. This figure provides the overall risk of bias across all studies. Left: this demonstrates the risk of bias assessment in four study domains. The highest risk of bias was in patient selection in view of the significant number of patients that were excluded due to insufficient imaging in most studies. Right: this demonstrates the risk of bias in applicability in three domains.


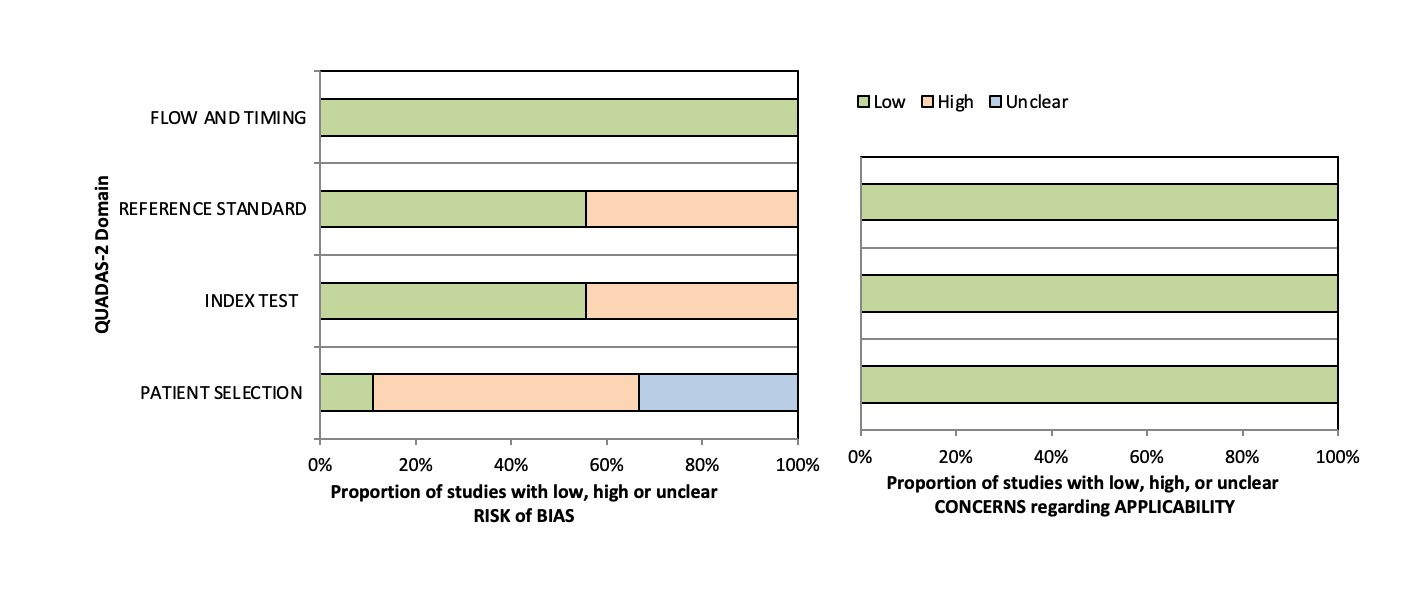


**Supplementary Figure 3. Publication bias**. This figure displays funnel plots to test for publication bias in the main study outcomes. The number of studies in each analysis was not sufficient to allow an adequately powered regression analysis to formally test funnel symmetry. A qualitative visual assessment was therefore used and there was minor funnel asymmetry for extent of resection (A), but not overall survival (B), progression-free survival (C) and *MGMT* (D).


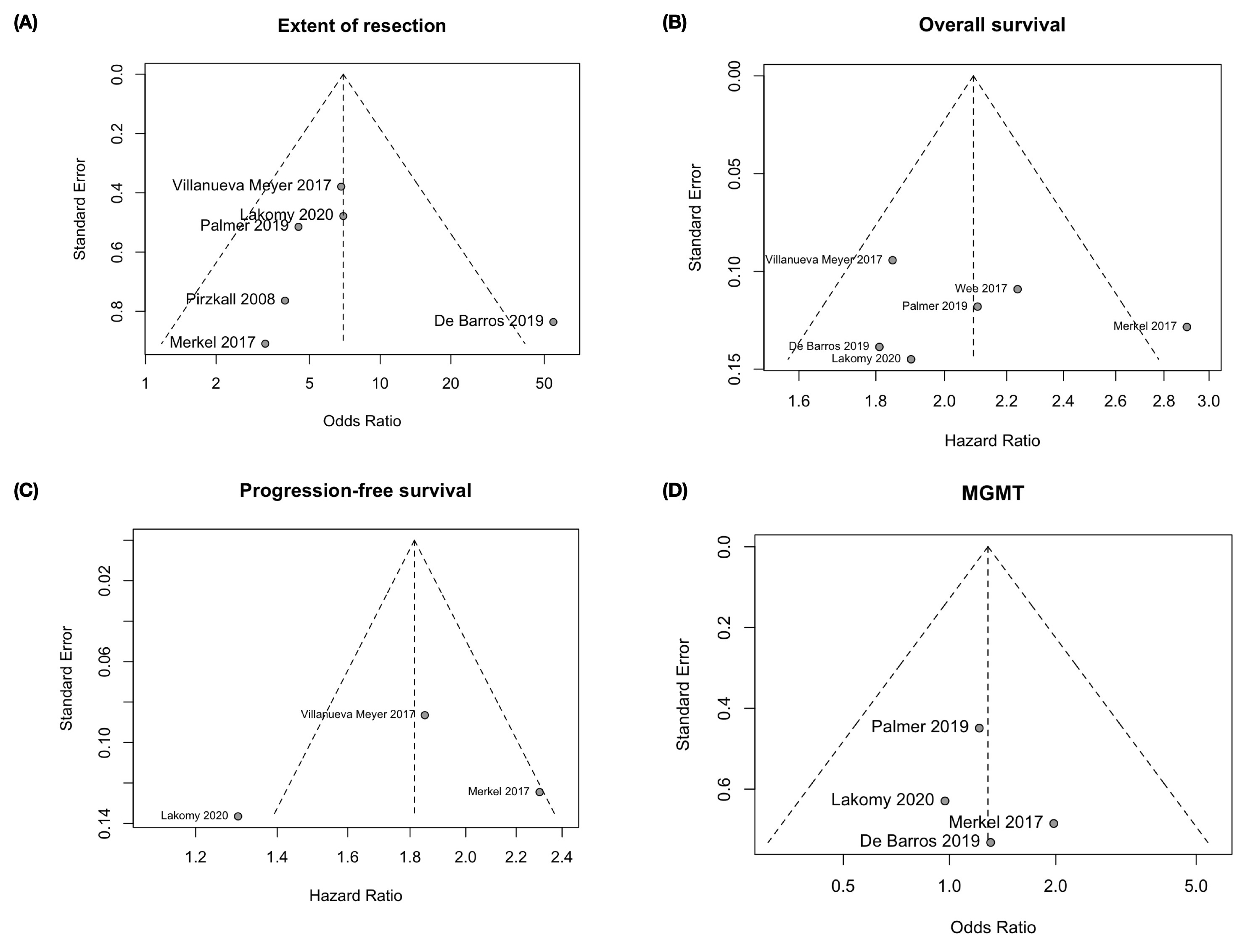


**Supplementary Figure 4. Sensitivity analysis.** This analysis was undertaken using a subset of studies that utilised functional imaging to define REP. Sufficient study numbers only permitted analysis of the variables shown. (A) Extent of resection – there was a higher incidence of REP after subtotal resection (OR 10.44, 95% CI 2.54-42.88, Z = 3.25, p = 0.001) using a random effects model. (B) Overall survival – REP was associated with a higher hazard ratio of death (HR 1.96, 95% CI 1.73-2.22, Z = 10.63, p < 0.001) using a fixed effects model.


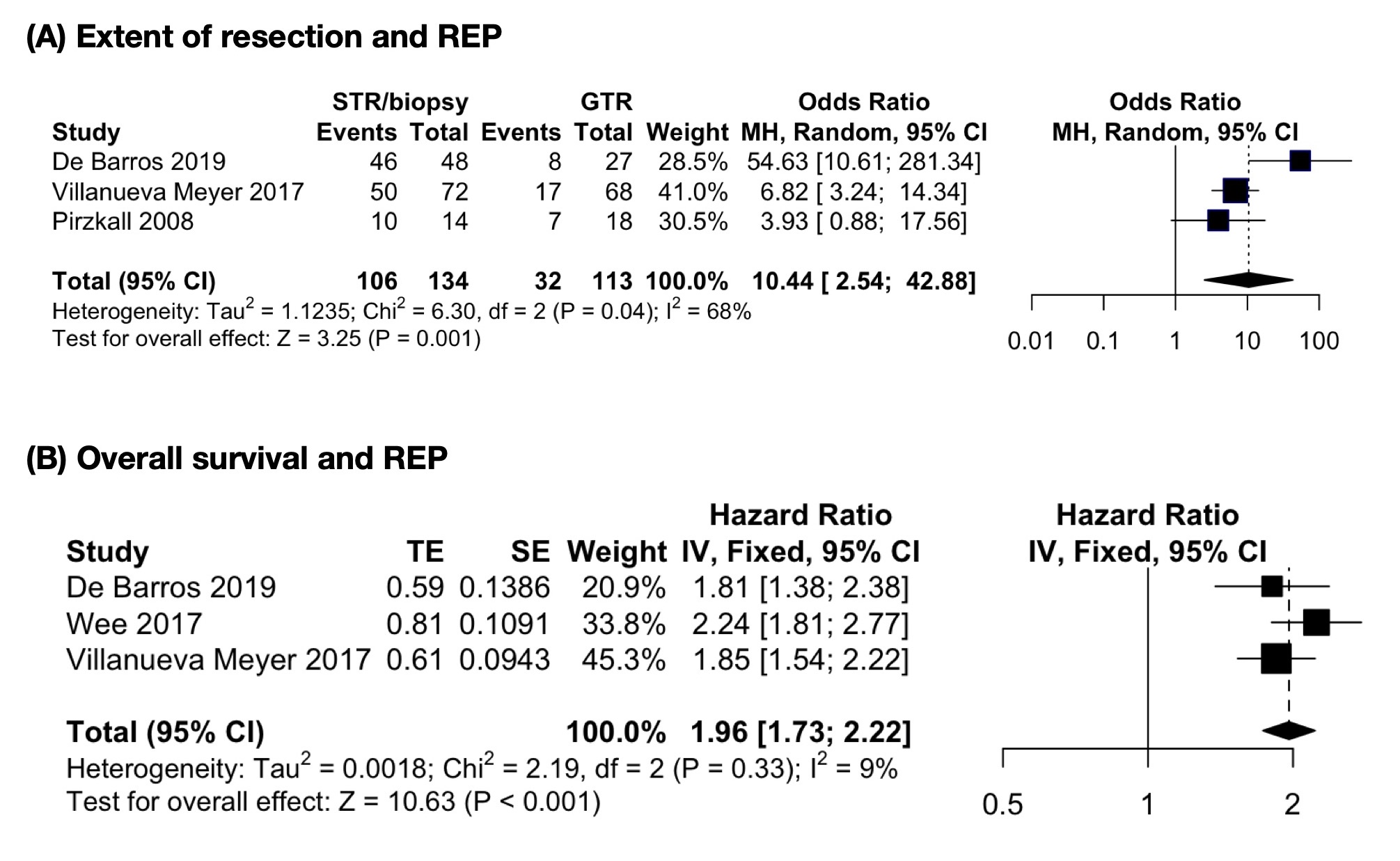

Supplement: vdac075_suppl_Supplementary_Figures [file vdac075_suppl_supplementary_figures.docx]
