# Supplementary material for: Rapid early progression (REP) of glioblastoma is an independent negative prognostic factor: Results from a systematic review and meta-analysis
Source: Neurooncol Adv. 2022 Jun 4;4(1):vdac075. doi: 10.1093/noajnl/vdac075 (PMC9234755; doi:10.1093/noajnl/vdac075)
Supplement: vdac075_suppl_Supplementary_Tables [file vdac075_suppl_supplementary_tables.docx]

**Supplementary Table 1. Literature search strategy.**

| **Search** | glioblastoma AND (progression OR recurrence)) AND (early OR rapid) |
| --- | --- |
| **Databases** | MEDLINE, Cochrane, EMBASE |
| **Time period** | All |

**Supplementary Table 2. PICOS table.** Further detail about outcomes is provided in the methods section.

| **Population** | Newly-diagnosed glioblastoma patients |
| --- | --- |
| **Intervention** | Comparison of postoperative and pre-radiotherapy MRI scans |
| **Comparator** | N/A |
| **Outcomes** | - Primary outcome: incidence of REP - Secondary outcomes:   - Impact of demographic factors and time between MRI scans on REP   - Impact of type of MRI scan sequences used to assess REP   - Impact of REP on overall survival (OS)   - Impact of REP on the location of future disease progression and progression-free survival (PFS)   - Impact of extent of resection on REP   - Impact of *MGMT* promoter methylation and IDH mutation status on REP |
| **Study design** | Any excluding case reports |

**Supplementary Table 3. Risk of bias assessment using the QUADAS-2 tool.** This table presents individual study data relating to risk of bias.

| **Study** | **RISK OF BIAS** | | | | **APPLICABILITY CONCERNS** | | |
| --- | --- | --- | --- | --- | --- | --- | --- |
|  | **PATIENT SELECTION** | **INDEX TEST** | **REFERENCE STANDARD** | **FLOW AND TIMING** | **PATIENT SELECTION** | **INDEX TEST** | **REFERENCE STANDARD** |
| Lakomy 2020 | ☹ | ☹ | ☹ | ☺ | ☺ | ☺ | ☺ |
| Palmer 2019 | ? | ☹ | ☹ | ☺ | ☺ | ☺ | ☺ |
| De Barros 2019 | ☹ | ☺ | ☺ | ☺ | ☺ | ☺ | ☺ |
| Merkel 2017 | ☹ | ☹ | ☹ | ☺ | ☺ | ☺ | ☺ |
| Wee 2017 | ☹ | ☺ | ☺ | ☺ | ☺ | ☺ | ☺ |
| Villanueva Meyer 2017 | ☹ | ☺ | ☺ | ☺ | ☺ | ☺ | ☺ |
| Majos 2016 | ☺ | ☹ | ☹ | ☺ | ☺ | ☺ | ☺ |
| Farace 2013 | ? | ☺ | ☺ | ☺ | ☺ | ☺ | ☺ |
| Pirzkall 2008 | ? | ☺ | ☺ | ☺ | ☺ | ☺ | ☺ |

☺Low Risk ☹High Risk ? Unclear Risk
